# Evaluation of methodologies for microRNA biomarker detection by next generation sequencing

**DOI:** 10.1080/15476286.2018.1514236

**Published:** 2018-09-18

**Authors:** Anna M.L. Coenen-Stass, Iddo Magen, Tony Brooks, Iddo Z. Ben-Dov, Linda Greensmith, Eran Hornstein, Pietro Fratta

**Affiliations:** a Sobell Department of Neuromuscular Diseases, UCL Institute of Neurology, London, UK; b Department of Molecular Genetics, Weizmann Institute of Science, Rehovot, Israel; c UCL Genomics, Institute of Child Health, London, UK; d Laboratory of Medical Transcriptomics, Department of Nephrology, Hadassah - Hebrew University Medical Center, Jerusalem, Israel

**Keywords:** microRNA, small RNA sequencing, biomarker, next-generation-sequencing, sequencing library preparation

## Abstract

In recent years, microRNAs (miRNAs) in tissues and biofluids have emerged as a new class of promising biomarkers for numerous diseases. Blood-based biomarkers are particularly desirable since serum or plasma is easily accessible and can be sampled repeatedly. To comprehensively explore the biomarker potential of miRNAs, sensitive, accurate and cost-efficient miRNA profiling techniques are required. Next generation sequencing (NGS) is emerging as the preferred method for miRNA profiling; offering high sensitivity, single-nucleotide resolution and the possibility to profile a considerable number of samples in parallel. Despite the excitement about miRNA biomarkers, challenges associated with insufficient characterization of the sequencing library preparation efficacy, precision and method-related quantification bias have not been addressed in detail and are generally underappreciated in the wider research community.

Here, we have tested in parallel four commercially available small RNA sequencing kits against a cohort of samples comprised of human plasma, human serum, murine brain tissue and a reference library containing ~ 950 synthetic miRNAs. We discuss the advantages and limits of these methodologies for massive parallel microRNAs profiling. This work can serve as guideline for choosing an adequate library preparation method, based on sensitivity, specificity and accuracy of miRNA quantification, workflow convenience and potential for automation.

## Introduction

microRNA (miRNAs) are short endogenous non-coding RNAs (ncRNA) with a central role in regulating post-transcriptional gene expression by modulating messenger RNA (mRNA) stability [1]. Interestingly, miRNAs have been detected in various biofluids including plasma or serum, and altered levels of circulating miRNAs were found to be indicative of tissue pathology [2,3]. Therefore, not only tissue miRNAs, but also extracellular miRNAs, have emerged as promising biomarkers to diagnose or monitor disease progression. Consequently, a significant effort has been devoted to studying cell-free microRNAs in various disease contexts as novel biomarkers [4,5]. Of note, blood or urine based biomarkers are especially desirable as these fluids are readily accessible and can be sampled repeatedly with minimally invasive procedures.

However, to comprehensively explore the biomarker potential of circulating miRNAs, accurate and cost-efficient miRNA profiling techniques are required. Next generation sequencing (NGS) does not rely on *a priori* gene annotation and thus allows for discovery of novel miRNA species [6]. Most importantly, NGS is sufficiently sensitive and cost-effective due to a high capacity for sample multiplexing. In addition, the single-nucleotide resolution provided by NGS enables the identification of isomiRs (miRNAs variants that differ in sequence or length from the annotated species in miRBase) [7]. Several studies have compared various miRNA profiling platforms such as microarrays, RT-qPCR or sequencing [8,9], however without assessing the procedure of library preparation itself, which has been shown to significantly influence the results obtained by sequencing [10–15]. There are significant challenges associated with small RNA sequencing library preparation, such as biased adapter ligation, formation of adapter dimers, the requirement to size-select the small RNA species, and the necessity to adapt for very low input protocols, especially if biofluids are analyzed. With increasing numbers of tools becoming available to prepare libraries for miRNA sequencing, there is an unmet need to characterise and compare these different methodologies for their efficacy and precision.

In order to generate small RNA sequencing (sRNA-seq) libraries, adapters are ligated to both ends of the miRNA, followed by reverse transcription, template amplification by PCR and size selection of small RNA species. Importantly, several of these steps have been shown to introduce biases and artefacts [10,14,16,17]. As such, certain adapter-miRNA pairs or sequence compositions may be favoured over others during ligation and PCR amplification, and thereby result in over- or underrepresentation of these miRNAs in the sequencing library [11,13]. Furthermore, the formation of adapter dimers or inefficient size-selection may impede enrichment for miRNAs over other RNA species and thus decrease the number of usable reads [11]. Lastly, a major challenge for the discovery of biofluid-based miRNA biomarkers is the necessity to work with very low input amounts of RNA.

In this study, we have benchmarked four commercial kits intended for Illumina sequencing: Nextflex Small RNA-Seq Kit v3, Bioo Scientific (BSC); SMARTer smRNA-Seq Kit, Clontech/Takara (CLT); NEBNext Small RNA Library Prep Set, New England Biolabs (NEB) and QIAseq miRNA Library Kit, Qiagen (QIA). All methodologies can generate miRNA sequencing libraries from low input amounts (1–100 ng) and are therefore suitable to process RNA derived from biofluids. Here, we prepared libraries from human plasma, human serum and murine brain tissue as well as from the synthetic RNA reference miRXplore (containing 950 miRNAs) for each kit in parallel. Of note, we did not include the frequently used TruSeq Small RNA Library Preparation Kit (Illumina), as the manufacturer does not recommend its usage for low input quantities. We aimed to critically evaluate the library preparation methodologies based on their i) sensitivity and accuracy for miRNA quantification; ii) capacity to enrich for miRNA mapping reads; and iii) convenience of workflow and potential for automation.

## Results

### Overview of the library preparation methodologies

**Figure 1.**
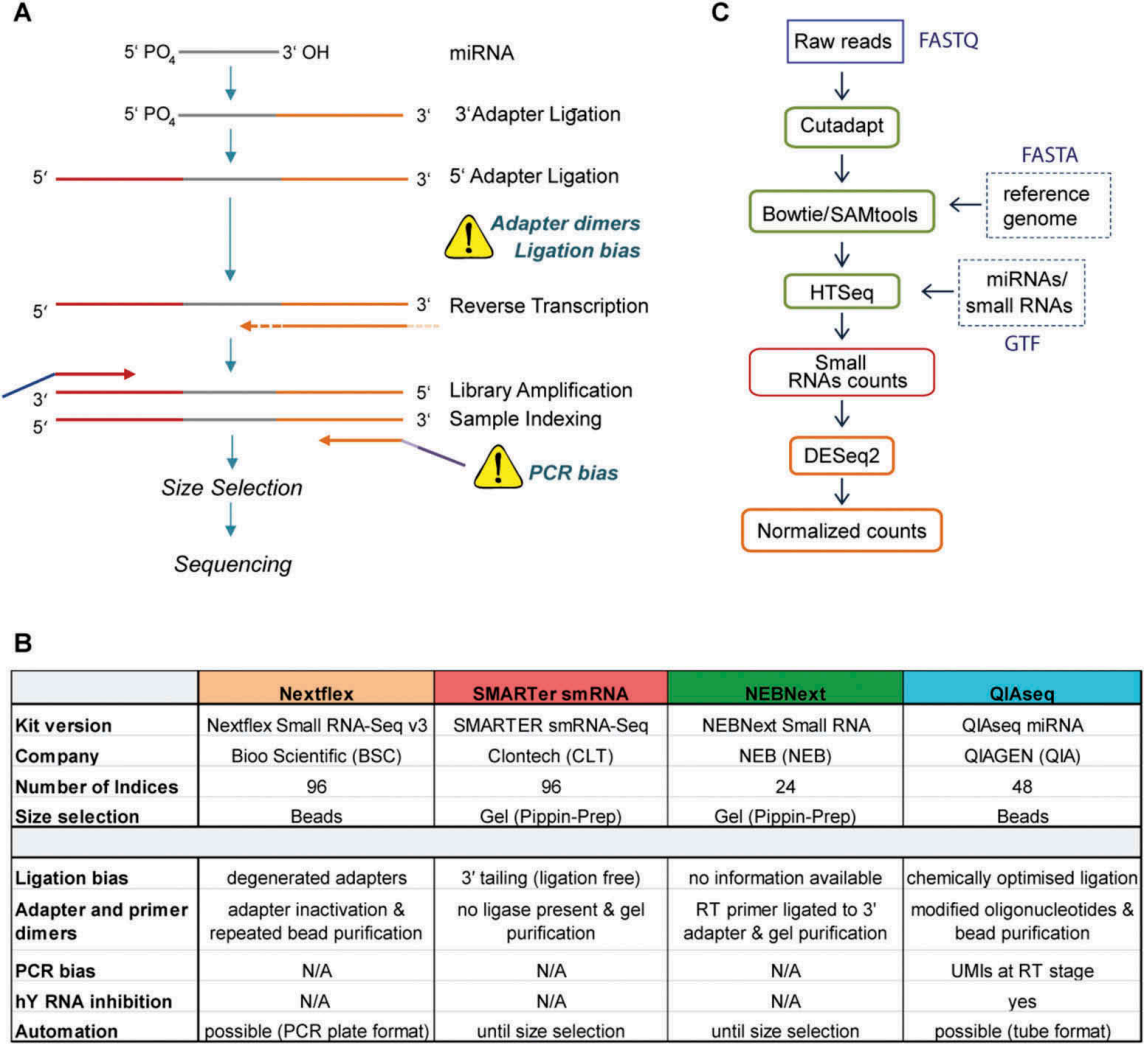
Overview of the small RNA sequencing workflow, compared methodologies for library preparation and computational analysis pipeline. (a) Schematic illustration of small RNA sequencing (sRNA-seq) library preparation. With exception of the SMARTer smRNA-Seq kit, all kits employed a ligation-based approach to attach 3′ and 5′ adapter to the miRNA. In the QIAseq protocol, a unique barcode is attached at the RT stage (orange dotted line), so-called unique molecular indices (UMI). Illumina adapter (purple and blue) and sequencing index (light purple) for multiplexing are added during the PCR amplification. Several steps of the protocol may introduce bias to the sequencing libraries as indicated by the yellow warning triangles. (b) Table summary of the key features of the four assessed sRNA-seq kits. (c) Small RNA sequencing data analysis pipeline. In the case of QIAseq, UMIs were trimmed alongside the adapters to allow side-by-side comparison by a single computational pipeline. Abbreviations: Nextflex Small RNA-Seq Kit v3 (Nextflex), NEBNext Small RNA Library Prep Set (NEBNext) and QIAseq miRNA Library Kit (QIAseq).

The typical workflow for preparation of small RNA sequencing (sRNA-seq) libraries is illustrated in Figure 1(a). Initially, adapters are attached to the 21–23 nt long miRNA to allow for PCR amplification and to accurately identify the native miRNA termini during sequencing. Subsequently, cDNA is generated, followed by PCR amplification, during which barcodes (to enable multiplexing) and sequencing index primers are introduced. After library amplification, amplicons of the correct size must be selected, for which typically either gel- or bead-based purification methods are used (Figure 1(a)).

The library preparation methodologies compared in this study have incorporated steps aimed to address quantification bias, adapter dimer formation and inefficient size selection (Figure 1(b)). Nextflex Small RNA-Seq Kit v3 (Nextflex), NEBNext Small RNA Library Prep Set (NEBNext) and QIAseq miRNA Library Kit (QIAseq) utilize RNA ligases to sequentially attach 3ʹ and 5ʹ adapters to the miRNA. Of note, the Nextflex kit features so-called degenerated adapters, where the last four nucleotides at the ligation junction are randomized with the aim of minimising sequence-dependent ligation bias [12,15]. Conversely, the SMARTer smRNA-Seq kit utilizes a ligation-free ‘tailing approach’, where initially the 3ʹ end is polyadenylated followed by a reverse transcription (RT) reaction primed by an oligo dT primer that incorporates the 3ʹ adapter. A specialized reverse transcriptase enzyme (PrimeScript™ Reverse Transcriptase) switches template upon reaching the end of each RNA template and utilizes the provided SMARTer smRNA-Seq oligo as secondary template to attach the 5ʹ adapter (all Clontech/Takara). All sequencing libraries were prepared strictly adhering to the manufacturer’s instructions (details in Material and Methods section and Table 1). Nextflex and QIAseq feature a gel-free size selection step, whereas SMARTer smRNA-Seq and NEBNext require gel separation. Following sequencing, raw reads were processed side-by side utilising the outlined computational pipeline (Figure 1(c)).

**Table 1.** Adapter dilutions and number of PCR cycles for sRNA-seq library preparation.

			BSC		CLT		NEB		QIA	
ID	Sample	Input (ng)	AD	PCR	AD	PCR	AD	PCR	AD	PCR
U1	miRXplore 1	5	1/1.3	16	N/A	13	none	15	none	15
U2	miRXplore 2	5	1/1.3	16		13	none	15	none	15
P1	hsa plasma 1	5	1/4	23		16	1/4	18	1/5	22
P2	hsa plasma 2	5	1/4	23		16	1/4	18	1/5	22
S1	hsa serum 1	5	1/4	23		16	1/4	18	1/5	22
S2	hsa serum 2	5	1/4	23		16	1/4	18	1/5	22
B1	mmu brain 1	200	1/1.3	16		13	none	15	none	15
B2	mmu brain 2	200	1/1.3	16		13	none	15	none	15

Abbreviations: miRXplore Universal Reference (U), human plasma (P), human serum (S), mouse brain (B), adapter dilution (AD).

### Assessment of miRNA quantification bias during library preparation

The miRXplore universal reference contains more than 950 synthetic miRNAs at equimolar ratio [18] and is therefore suitable to assess distortions of miRNA counts that are introduced during library preparation. Of note, previous studies have carefully validated the universal reference utilizing spike-in strategies [19], and miRXplore has been widely used in both microarray and RNAseq studies [13,20–22]. Here we aimed to compare the capability of each kit to capture miRNA abundance independent of sequencing performance or depth. Consequently, equal numbers of mapped reads (sampled by using 570,000 mapped reads in random order) were aligned and counted for each kit (count statistics are summarized in Supplementary Table S1). To enable parallel analysis of the four kits utilizing the same analysis pipeline, we did not apply the UMI normalization offered by QIAseq.

**Figure 2.**
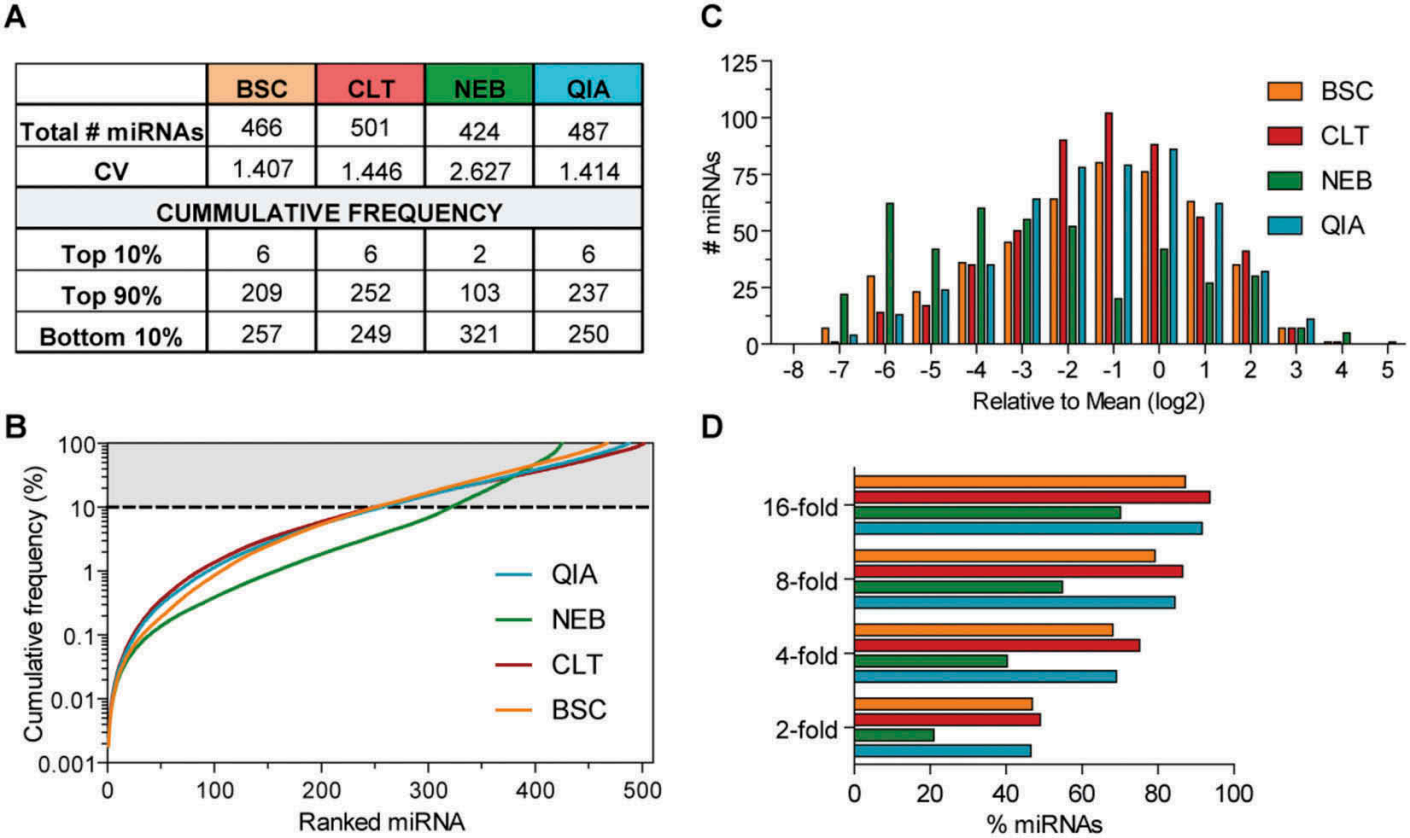
Diversity and dynamic range of sRNA-seq libraries utilising a synthetic miRNA reference. Parallel testing of the sRNA-seq library preparation methodologies against the miRXplore universal reference (Miltenyi Biotec) library containing 539 synthetic miRNAs of human origin (*n *= 2 technical replicates). (a) Table summarising sensitivity and library complexity. The number of detected microRNAs with > 10 reads (using mean counts of the replicates) and the coefficient of variation (CV) were determined. (b) Cumulative frequency plot of the mean miRNA counts of the replicates for each methodology. The number of miRNAs that comprise the top 10%, the top 90% (where top means most abundant), and the bottom 10% of all miRNA reads (least abundant) for each methodology are listed in the table (a). The grey shaded area corresponds to miRNAs accounting for the top 90% of reads. (c) Histogram showing the log2 transformed ratio of individual miRNA count versus the mean count of all miRNAs. (d) Bar chart depicting the percentage of miRNAs within x-fold of the mean.

Of the 539 human miRNAs in the reference, on average, 470 distinct miRNA species were detected (mean count of the replicates > 10 reads). The number of miRNA species identified by the kits was comparable although SMARTer smRNA-Seq kit detected the most miRNAs (501), followed by QIAseq (487), Nextflex (466) and NEBNext (424) (Figure 2(a)). The number of identified miRNAs is an indicator for sensitivity and the capacity of the library preparation methodology to reflect the true diversity of the input material.

Due to the equimolar abundance of each synthetic miRNA in the reference, all detected miRNAs are expected to be sequenced with equal counts. The relative variability in data sets, calculated as the ratio of standard deviation to the mean, and known as the coefficient of variation (CV), was similar in Nextflex, SMARTER smRNA-Seq, QIAseq (~ 1.4), indicating a comparable performance. In contrast NEBNext libraries showed a higher CV (~ 2.7) (Figure 2(a)). Likewise, plotting the cumulative frequency of miRNA counts against ranked miRNAs, revealed that a larger number of miRNA species was detected with similar counts (as indicated by a flatter slope of the curve) by Nextflex, SMARTER smRNA-Seq and QIAseq than with the NEBNext kit (Figure 2(b)).

Library complexity can also be assessed by the number of miRNAs accounting for the top 90% of the total read counts. The complexity of SMARTER smRNA-Seq library was highest as 252 miRNAs comprised the upper 90% of read counts, followed by QIAseq (237), Nextflex (209) and NEBNext (103). Additionally, we also generated histograms to capture the number of miRNAs within x-fold of the mean (Figure 2(c,d)). The abundance of 45–49% of detected miRNAs were within 2-fold of the mean in libraries prepared with Nextflex, SMARTER smRNA-Seq or QIAseq, whilst NEBNext detected only 20% within 2-fold (Figure 2(d)). Overall, SMARTER smRNA-Seq and QIAseq provided the most accurate and sensitive miRNA quantification in the synthetic reference sample.

Interestingly, the top 10% of total miRNA reads was accounted for by only 2–6 miRNAs for all kits. To determine possible common molecular features of the overrepresented miRNAs, we used the DREME tool from the MEME suite to discover short sequence motifs [23]. However, no significantly enriched motifs were found for any of the methodologies (*P*-value adjusted > 0.05 (data not shown)), therefore providing no evidence for specific sequence motifs or nucleotide composition to cause the overrepresentation of some synthetic miRNAs.

Furthermore, we systematically correlated the mean read count for each library preparation methodology with mean read counts of the other kits using Pearson correlation analysis to detect similarities between the methodologies (Table 2). Although significant *p*-values were determined for all comparisons, the correlation coefficient *r* indicated only weak or modest correlation (ranging from 0.228–0.554), suggesting that a large amount of the observed count data could not be explained by the correlation analysis. Interestingly, the highest correlation coefficient was determined for Nextflex and QIAseq (0.554) which utilize adapters of similar sequences. However, we could not detect any sequence motifs in miRNAs commonly overrepresented in these two methodologies using DREME.

**Table 2.** Correlation analysis of miRNA abundance in the miRXplore universal reference.

*r*	BSC	CLT	NEB	QIA
BSC	1.000	0.387	0.359	0.554
CLT	0.387	1.000	0.228	0.459
NEB	0.359	0.228	1.000	0.402
QIA	0.554	0.459	0.402	1.000

Pearson correlation coefficient (*r*) as indicated. All *p-*values < 0.01.

Lastly, we randomly selected three miRNAs present in the miRXplore reference to validate their abundance using RT-qPCR as an orthologue method (Supplementary Figure S1). We detected all of three miRNAs with less than 1.5 fold change to the mean, whereas in some sequencing libraries, these miRNAs were detected to divert from the mean expression by up to 5 fold. These results confirm that although these miRNAs were found to be similarly abundant in RT-qPCR, each library preparation methodology distorted miRNA abundance in a unique pattern.

### Small non-coding RNA biotype distribution in serum and plasma

Next, we carried out library preparation on plasma and serum samples derived from healthy volunteers (*n *= 2) using all four protocols. Notably, both plasma and serum are a rich source of different classes of small ncRNAs (full-length RNA and fragmented variants) [24]. Consequently, to maintain sequencing depth while multiplexing large numbers of samples, a library preparation methodology should enrich miRNAs over other small ncRNAs and mRNA degradation products.

**Figure 3.**
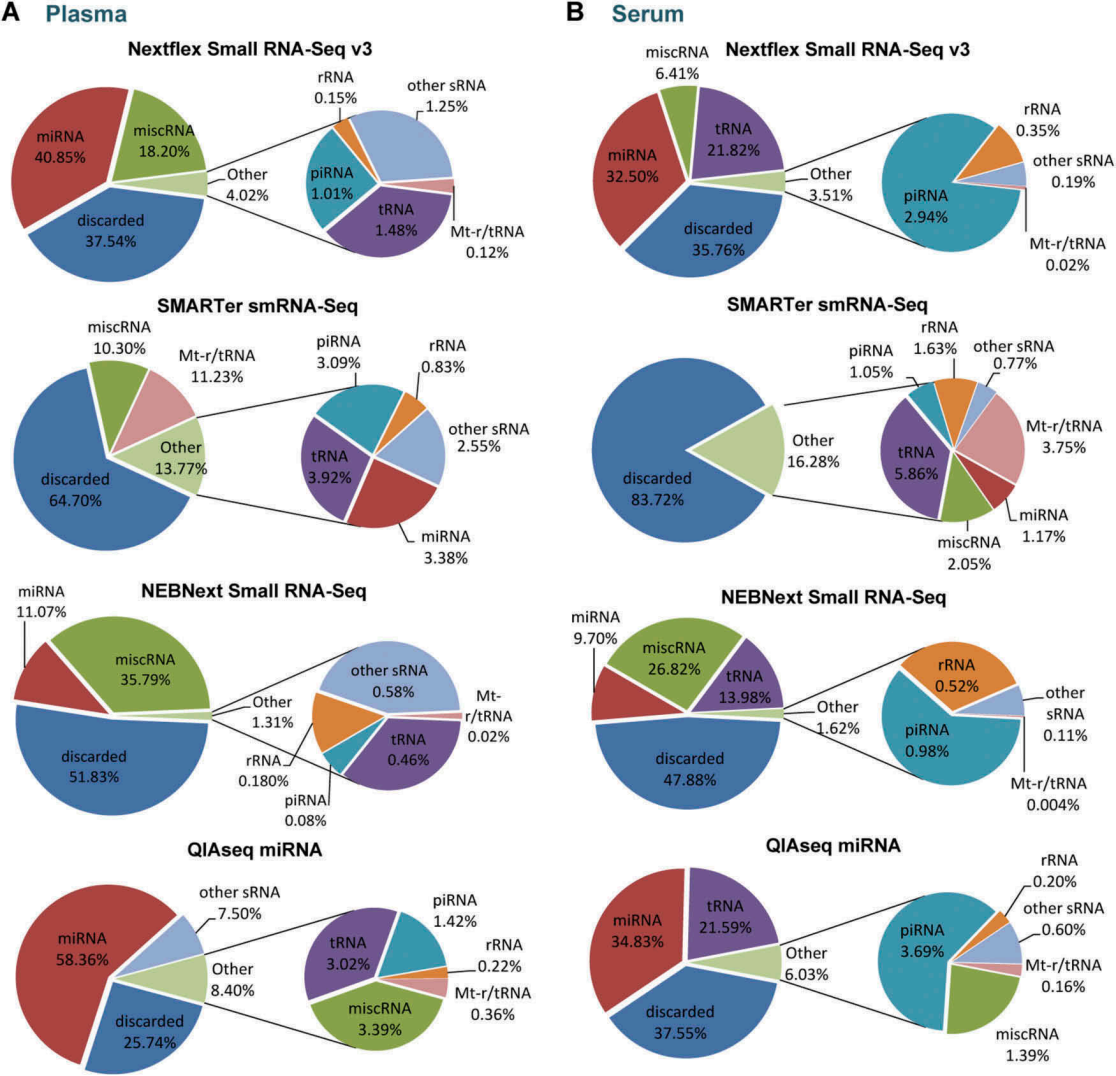
Small RNA library composition by biotype for human plasma and serum samples. Pie charts analysis of the mean percentages of mapped sRNA reads from (a) human plasma and (b) human serum. Mapped reads were sorted into the following ncRNA biotype classes: miRNA, tRNA, rRNA, piRNA, mt-t/rRNA, other sRNA (snoRNA, snRNA, scaRNA and scRNA) and miscRNA (vt-RNA and YRNA). Mapped reads that did not align to ncRNA genes in the human genome were classified as ‘not aligned’. Lowly abundant sRNAs biotypes were combined in the charts as ‘Others’. *n *= 2 biological replicates for plasma and serum respectively, pie charts show mean percentage.

We sorted the mapped reads into the following biotypes of small ncRNAs: miRNA, transfer RNAs (tRNA), ribosomal RNAs (rRNA), piwi-interacting RNAs (piRNA), mitochondrial transfer and ribosomal RNAs (mt-t/rRNA), other small RNAs (sRNA) (including small nucleolar RNAs (snoRNA), small nuclear RNAs (snRNA), small cajal body-specific RNAs (scaRNA) and small cytoplasmic RNAs (scRNA)), miscellaneous small RNAs (miscRNA) (including vault RNA (vt-RNA) and Y RNAs (YRNA)) and ‘not aligned’, referring to mapped reads that did not align to small ncRNA genes in the human genome (Figure 3; Supplementary Table S2 and S3). The percentages of mapped reads corresponding to miRNAs were as follows: QIAseq (~ 58% in plasma, 35% in serum), Nextflex (~ 41% in plasma, ~ 33% in serum), NEBNext (11% in plasma, 10% in serum) and SMARTer smRNA-Seq (3% in plasma, 1% in serum). These data suggests that QIAseq and Nextflex provide higher miRNA enrichment relative to the two other tested kits. Of note, the percentage of miRNA mapping reads was consistently higher in plasma compared to serum for all methodologies.

A considerable number of reads (65% for plasma, 84% for serum) from the libraries prepared with SMARTER smRNA-Seq were discarded since they did not align to any small RNA in the annotated reference file (Figure 3; Supplementary Table S1 and S2). Given the fact these reads were successfully mapped to the genome, they are likely to correspond to other RNA species. One potential reason for reduced selectivity of the SMARTer smRNA-Seq kit is that it does not exclude mRNA degradation products whereas library preparations with all other kits require an intact 5ʹ phosphate and 3ʹ hydroxyl groups for adapter ligation [25]. Accordingly, the TapeStation profile of SMARTer smRNA-Seq revealed a broad spectrum of RNA molecules of a larger size than miRNAs prior to size-selection. In contrast, the profile of NEBNext before size-selection was already enriched in miRNA species (Supplementary Figure S2). Overall, libraries prepared from plasma were more enriched for miRNAs compared to matching serum libraries and overall, QIAseq and Nextflex were most efficient in sequencing miRNAs in both biofluids.

Besides miRNAs, tRNA derived fragments (tRFs) were frequently detected in serum (16% of mapped reads, averaged across all libraries) but less abundant in plasma (2%). In contrast, the amount of YRNA derived fragments (grouped under miscRNA) mapping reads was typically higher in plasma (17%) compared to serum (10%) (Figure 3). Interestingly, 87–99% of miscRNAs in human plasma corresponded to human YRNA species consistently across all kits (data not shown). QIAseq employs a chemical blocking strategy to prevent this RNA species from incorporation into the sequencing library which may be responsible for the observed reduction in reads mapping to miscRNA in the QIAseq libraries (less than 4% in all samples).

### miRNA signatures in serum and plasma

**Figure 4.**
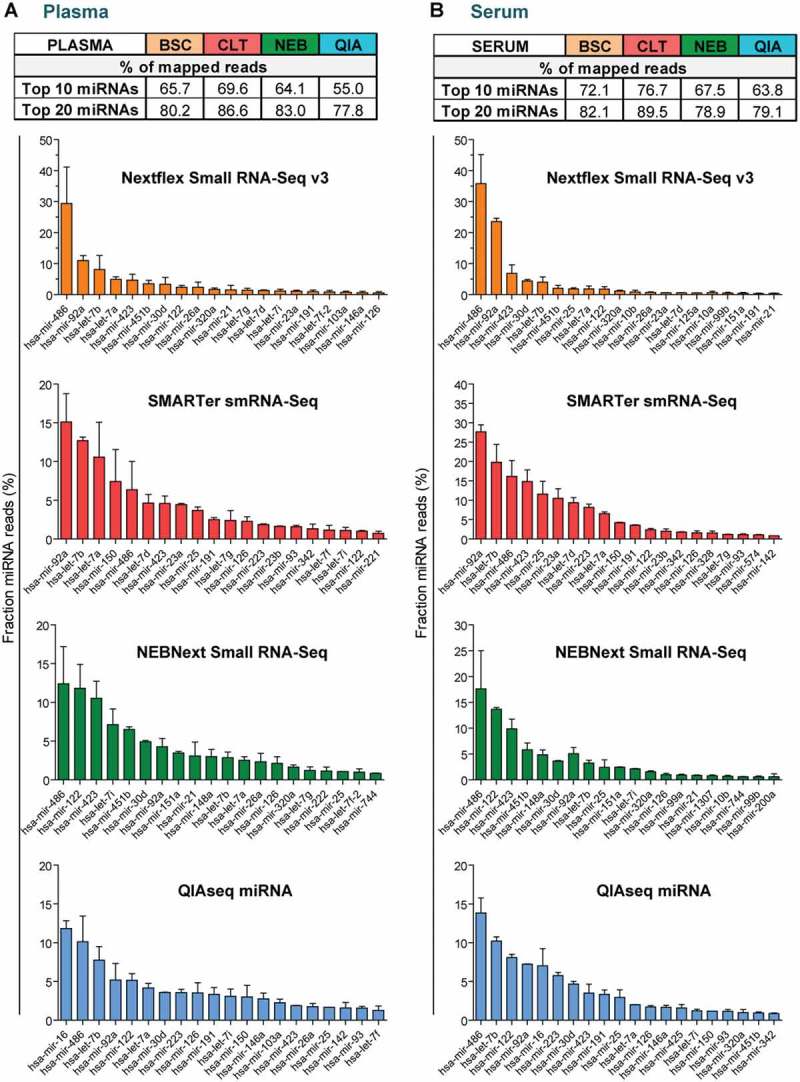
miRNA signatures in human plasma and serum samples. Overview of the miRNAs with the highest count numbers in each library for (a) human plasma and (b) human serum. The table indicates the percentage of sequencing reads accounting for the top 10 or top 20 most abundant miRNAs respectively (mean normalized frequencies used). Bar charts indicate the mean percentage of reads associated with the 20 most abundant miRNAs separated by biofluid and kit. Typically 10 miRNAs were accounting for more than 55% of all miRNA mapping reads. Most libraries were enriched for miRNAs that are typically found in blood-derived samples, such as miR-486. *n *= 2, values are mean + SD.

We next sought to investigate the miRNA composition of the plasma and serum libraries. Normalized counts were ranked by ascending abundance and the cumulative frequency (using the mean count of the replicates) was calculated. Previous studies have reported an asymmetric distribution of miRNAs in biofluids and tissues (resembling a Pareto distribution), wherein a small number of miRNAs accounts for the majority of total reads [24,26,27]. In line with these findings, the ten most frequently detected miRNAs accounted for 64% of all miRNA mapping reads in plasma and 70% in serum samples (averaged across all kits) (Figure 4(a,b)). For libraries prepared with QIAseq, this percentage was lower than the average (55.0% in plasma and 63.8% in serum), indicating a slightly less skewed distribution of miRNA counts.

In order to determine whether the observed biofluid miRNA signature was in concordance with previously published studies, the normalized read frequency of the 20 most abundant miRNAs was plotted for each methodology in descending abundancy (Figure 4(a,b)). Interestingly, 8 of the 20 most abundant miRNAs in plasma, and 6 of the 20 most frequently detected miRNAs in serum, were common across all methodologies (Supplementary Figure S4(a,b)). This considerable degree of overlap highlights that despite different library preparation methodologies, clear similarities in miRNA signatures could be detected. Most of the commonly detected miRNAs have been described previously to be highly abundant in blood-derived samples e.g. miR-486, miR-92a, miR-423 and members of the miR-let-7 family [24,26,28]. In summary, all assessed kits were enriched in miRNAs typically in blood and several of those miRNAs were commonly detected by multiple methodologies.

### Small non-coding RNA biotype distribution and miRNA signatures in murine brain

**Figure 5.**
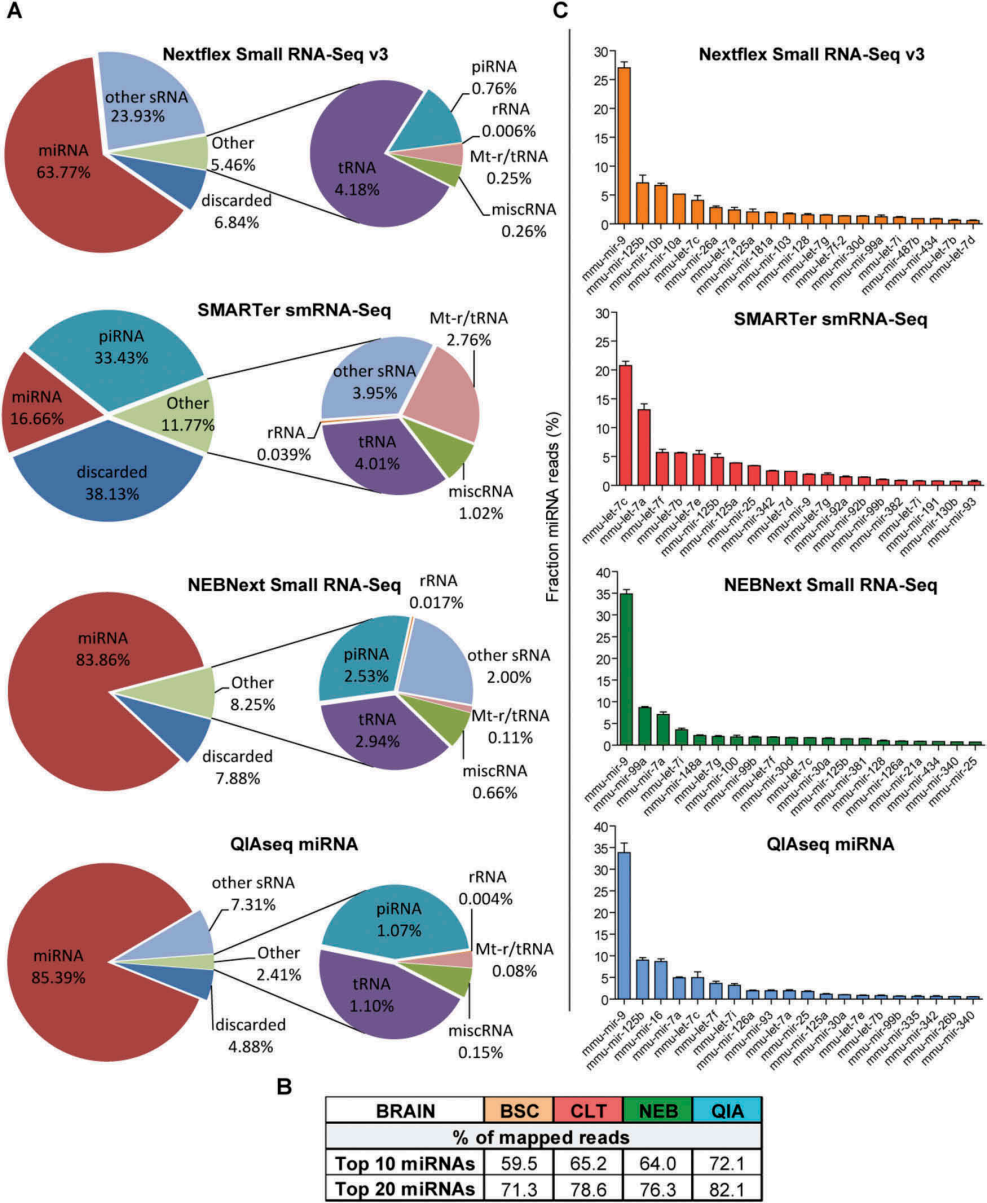
Small RNA library compositions by biotype and miRNA signatures of murine brain samples. Pie chart analysis of the mean percentages of small RNA reads from murine brain tissues. Mapped reads were sorted into the following ncRNA biotype classes: miRNA, tRNA, rRNA, piRNA, mt-t/rRNA, other sRNA (snoRNA, snRNA, scaRNA and scRNA) and miscRNA (vt-RNA and YRNA). Mapped reads that did not align to ncRNA genes in the human genome were classified as ‘not aligned’. Lowly abundant sRNAs biotypes were combined in the charts as ‘Others’. Pie charts depict mean percentage of each sRNA biotype relative to mapped reads. (b) The table indicates the percentage of sequencing reads accounted for by the top 10 or top 20 most abundant miRNAs (mean normalized frequencies used). (c) Bar charts indicate the mean percentage of reads associated with the 20 most abundant miRNAs in each library. *n *= 2 biological replicates, values are mean + SD.

To assess the capacity of each library preparation methodology for miRNome profiling of tissue samples, we prepared libraries from murine brain tissue. In contrast to plasma and serum, libraries generated from murine brain tissue, with exception of the SMARTer smRNA-Seq kit, contained mainly miRNA-mapping reads (Figure 5(a)). The percentage of mapped miRNA reads was as follows: 85% for QIAseq, 84% for NEBNext, 64% Nextflex and 17% for and SMARTer smRNA-Seq (Figure 5(a), Supplementary Table S4). Some libraries contained considerable amounts of other small RNA species, such as snoRNAs (~ 30% in NEXTflex samples and 7% in QIAseq samples). Similar to the plasma and serum, a substantial proportion of the reads for the SMARTer smRNA-Seq did not align to ncRNA genes and 40% of the reads were thus discarded (Figure 5(a), Supplementary Table S4).

Overall, the miRNA distribution in the tissue libraries was uneven and the top 10 most abundant miRNAs accounted for 65% of all miRNA-mapping reads (utilising mean normalized read frequency) (Figure 5(b.c)). Libraries prepared with Nextflex, NEBNext and QIAseq were highly enriched in miR-9, a miRNA abundant in brain with an established role in neurogenesis [29,30], (Figure 5(c)). While miR-9 was also abundant in SMARTer smRNA-Seq libraries (rank 11), members of the let-7 family were detected with higher frequency. Notably, published miRNA sequencing data confirm miR-9, several members of the let-7 family, as well as miR-125 and miR-30 as being highly abundant in murine brain tissue^,^ [31]. This consistency with previous studies indicates that all methodologies generated miRNA signatures typical for brain tissue, albeit the overlap across the four kits was slightly less compared to the biofluids (Supplementary Figure S4(c)). In general, small RNA libraries generated from tissue samples are more enriched in miRNAs compared to blood, and in particular, the QIAseq and Nextflex libraries were comprised of more than 80% miRNA mapping reads.

### Sequencing efficiency of libraries prepared from biofluids and brain tissue

**Figure 6.**
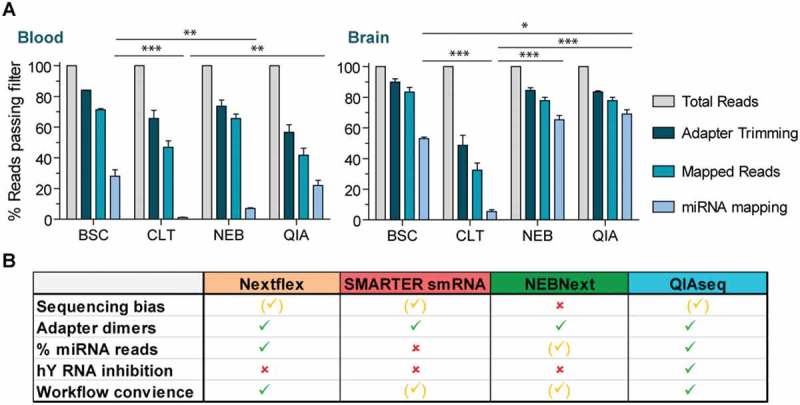
Summary of sequencing efficiency and overall performance. (a) To assess sequencing efficiency, the percent of reads passing the filter during adapter trimming and mapping were calculated for each of the four library preparation methodologies. The bar graph indicated the average percentage relative to total reads (corresponds to 100%) for biofluids (combing plasma and serum) and brain libraries. Values are mean + SEM, *n *= 4 for biofluids, *n *= 2 for brain. *P < 0.05, **P < 0.01, ***P < 0.001, significance only shown for miRNA mapping reads, one-way ANOVA with Bonferroni post hoc test. Values are mean + SD(b) Summary of the comparison for the sRNA-seq library preparation protocols. Green ticks indicate a satisfying and, yellow ticks an average performance in the respective category. Red crosses either signify a performance below average or in the case of YRNA inhibition, the option is not available.

During analysis of RNA sequencing experiments, the filtering of reads mainly occurs during removal of adapters (reads without adapter or too short reads) and mapping (unmapped or multi-mapped reads; in our study reads mapping to the reference genome more than 20 times were discarded). The number of reads passing these filters indicates the proportion of reads which are usable for downstream analysis and as such, gives an indication on sequencing efficacy. We calculated the percentage of reads passing these two initial filtering steps for the four library preparation methodologies for human blood (averaged across plasma and serum libraries) and murine brain tissue (Figure 6(a)). The number of usable reads was typically higher in tissue libraries compared to blood, with exception of the SMARTER smRNA-Seq kit. This finding is likely a consequence of an increased abundance of fragmented RNA species in the circulation, which may interfere with the enrichment for miRNAs. In addition, we investigated the presence of adapter dimers both visually and by bioinformatics analysis (Supplementary Figure S3). No adapter dimers were observed by visual inspection of the library size profiles, with exception of the NEBNext libraries, in which a small number of adapter dimers was detected by visual and computational analysis (corresponding to 0.27% of total reads).

In the case of miRNA biomarker discovery, the percentage of reads mapping to miRNAs is of highest relevance and we therefore focused the statistical analysis on this proportion of reads. After combining the data for plasma and serum, the proportion of raw reads corresponding to miRNAs was as follows: 28% for Nextflex, 22% for QIAseq, 7% for NEBNext and 1% SMARTer smRNA-Seq (Figure 6(a)). The performance of the kits and their comparison is different based on whether these are applied to peripheral biofluids or tissue. Consequently, if human blood is profiled, Nextflex and QIAseq provide a significant higher enrichment for miRNA mapping reads relative to the two other tested kits. In the case of murine tissue, QIAseq and NEBNext performed best, with 69% and 65% of the total reads aligning to miRNAs, respectively. For QIAseq, this was a significant increase in miRNA enrichment compared to Nextflex (53%) and SMARTer smRNA-Seq (6%) (Figure 6(a)).

In conclusion, Nextflex and QIAseq allow for efficient miRNome profiling in both blood and brain tissue, while NEBNext delivered an equivalent performance in brain tissue but not in biofluid samples. Libraries generated by the SMARTer smRNA-Seq displayed less enrichment of miRNA mapping reads, suggesting that a considerably higher sequencing depth would be required to achieve comparable miRNome coverage.

## Discussion

In this study we have undertaken a comprehensive comparison of four commercially available small RNA sequencing methodologies applicable to low-input material (Figure 1). In recent years, evidence has emerged that during adapter ligation, some adapter pairs or sequence compositions may be favoured over others. The consequence is a distortion of miRNA quantity from the true abundance in the sample, thus resulting in over- or underrepresentation of certain miRNAs in the sequenced libraries [10,14]. A study using identical starting material but different methodologies to generate libraries for sequencing, demonstrated that miRNA expression may vary up to four orders of magnitudes between datasets [14]. The observed bias was largely independent of sequencing platforms, but strongly correlated to the methodology employed to prepare the small RNA libraries [14,16]. Importantly, variation in the choice of RNA ligase, substrates (miRNA and adapter sequence as well as secondary structure and RNA-adapter co-folding) and reagents are all likely to account for some of the observed biases (reviewed in detail in [11]). Several studies suggest that the introduction of 3–5 randomized nucleotides to the ligation boundaries, as incorporated into the Nextflex adapters, alleviates adapter ligation bias and increases library diversity [12,32]. Similarly, approaches bypassing a ligation step by using 3′-end tailing by polyadenylation (as employed by the SMARTer smRNA-Seq kit) have been developed, although polyadenylation may also be subjected to bias, for example due to primary and secondary structure of the RNA substrate [11].

Here, we initially focused on assessing systematic bias introduced by the library preparation protocol using the universal reference miRXplore. Although miRNAs are represented in this reference at an equimolar ratio, only between 20–45% of detected human miRNAs were quantified within 2-fold of the miRNA population mean (Figure 2). SMARTer smRNA-Seq, Nextflex and QIAseq exhibited very similar CVs, cumulative frequency plots, as well as an almost identical number of miRNAs detected within 2-fold of the mean (Figure 2). Accordingly, utilising either a ligation-free technology or adapters with degenerated ends (random nucleotides) did not confer a significant improvement in accuracy of miRNA quantification, at least if benchmarked to QIAseq (direct ligation of adapters to miRNAs). However, these three methodologies demonstrated a superior performance compared to the NEBNext kit, which likewise relies on direct ligation of the adapter to the miRNA. Given the observed results with QIAseq, it appears that careful optimization of ligation enzyme, adapter sequence and reaction conditions may alleviate bias to similar extent as degenerated adapters or 3ʹ-end tailing. Overall, with a maximum of 49% of detected miRNAs quantified within 2-fold of the mean, our results suggest that despite advances, a significant distortion remains for many of the detected miRNAs in the synthetic reference (Figure 6(b)). Given that no enriched sequence motifs were found in the overrepresented synthetic miRNAs for any of the kits, we were unable to draw conclusions on potential molecular features causative of skewed miRNA quantification.

Of note, besides adapter ligation, clonal amplification of the input material during PCR represents another potential source of bias. To this end, the QIAseq kit features unique molecular indices (UMIs), which are introduced at the RT stage to address this source of bias. Whereas this technology may be especially useful if many PCR cycles are required to generate enough material for sequencing, it only accounts for bias that occurs after adapters have already been ligated. To this end, multiple studies suggest that the bias conferred by clonal amplification during PCR is negligible compared to bias created during adapter ligation [12,13,25]. Qiagen provides the GeneGlobe data analysis tool that is capable of UMI deconvolution and sRNA mapping, but we chose not to exploit this tool as the aim of our study was to undertake a parallel comparison utilising the same computational pipeline for all kits. This is of particular importance in the case of miRNA sequencing, where reads are short and often map to multiple locations in the genome. In this case, the choice of mapping and counting tool, supplied parameters and annotation files inevitably impacts the results. In addition, GeneGlobe employs a sequential mapping strategy, during which reads are first mapped to mature miRNAs annotated in miRBase version 2.1, then unmapped reads are further sequentially mapped to miRNA hairpins, piRNAs, tRNAs, rRNAs, mRNAs and other RNAs. Consequently, a direct comparison of QIAseq libraries analyzed with GeneGlobe and other library kits analyzed by an alternative computational pipeline is difficult. As a result, it is beyond the scope of this study to comment on utility of UMIs and the GeneGlobe analysis platform.

Further challenges of the adapter ligation step are intramolecular circularization reactions or the formation of adapter dimers as side products. The latter may result in contamination of the miRNA sequencing libraries, thus decreasing the sequencing depth by reducing the number of usable reads (Raabe et al. 2014). The methodologies tested in this study use chemically pre- adenylated 3ʹ adapters to prevent adapter circularization and employ various methodologies to avoid formation of adapter dimers (Figure 1(b)). In our comparison study, very few adapter dimers were observed in the NEBNext, an even smaller amount in the QIAseq kit and negligible quantities in the other two kits, highlighting that overall all methodologies sufficiently inhibited adapter dimer formation (Supplementary Figure S3 and Figure 6(b)).

Another important criterion to select a library preparation protocol for miRNA profiling is the percentage of reads that pass filtering steps and subsequently map to miRNAs, especially if many samples are sequenced in parallel and sequencing depth is critical. In this study, we found that in the case of plasma and serum, libraries prepared with QIAseq or Nextflex were most enriched for miRNA reads, whereas in brain tissue, QIAseq and NEBNext performed best (Figures 3, 5 and 6(a,b)). Although we only tested murine brain tissue, we hypothesise that other tissues of murine or of species with related origin will generate similar results with respect to sequencing efficiency and small RNA biotype composition. Nevertheless, our study has the limitation of having analyzed only selected sample types that we considered to be very commonly used in miRNA biomarker and discovery studies.

Of note, we found that miRNA mapping reads were more enriched in plasma compared to serum across all kits, suggesting plasma may be the more resourceful biofluid for circulating miRNA profiling. Furthermore, we consistently detected a considerable amount of tRFs in the serum samples, whereas in the plasma libraries YRNA fragments were much more frequently detected. Possible explanations for this asymmetry could either be that tRFs are preferentially released by blood cells during the coagulation process or alternatively, that tRFs are destabilised by clotting activators such as EDTA [33].

Finally, if sRNA-seq is used as a profiling method for large numbers of samples, a fast and automated protocol desirable. To this end, SMARTER smRNA-Seq features the quickest workflow up until the gel-based size-selection step which requires more time and labour even if the Pippin system is used. Importantly, both Nextflex and QIAseq offer the possibility to complete the entire workflow on one robotic platform since size selection is carried out in tubes utilising magnetic bead separation. We found handling of the Nextflex kit slightly more convenient as the complete library preparations can be performed in 96-well format. Conversely, QIAseq requires a larger volume for bead separation and therefore necessitates a switch to 1.5 ml tubes, thus reducing the number of samples processed in parallel (Figure 6(b)). In contrast, the Nextflex workflow includes one additional step of bead separating compared to QIAseq, thereby increasing samples processing time.

In summary, all protocols assessed in this study successfully generated libraries from low amounts of RNA derived from complex biofluids, although the number of miRNAs mapping reads varied considerably across RNA sources and library preparation methodologies. Consequently, depending on source of input material and the aim of the study (sometimes inclusion of other small RNA fragments, such as tRFs might be desired), the library preparation methodology of choice may differ. Nevertheless, we found that Nextflex and QIAseq consistently performed well with respect to enrichment of miRNA mapping reads in biofluids and tissues. Furthermore, both kits exhibited a relatively low quantification bias and have the capacity to be automated for high-throughput miRNA library preparation. Overall, our study highlights that despite various improvements in library preparation methodologies, miRNA quantification bias remains. Given that most miRNA biomarker studies focus on the discovery of differentially expressed miRNAs, it is important to highlight that quantification bias *per se* does not compromise the ability to detect expression changes if control and disease samples are processed in parallel. However, severe quantification bias reduces the number of reads available to lowly abundant miRNAs, thus consequently impacting the capacity to reliably detect changes in their abundance. In this case, deeper sequencing or selecting a kit with less skewed miRNA quantification may be advisable.

In conclusion, small RNA sequencing remains challenging despite considerable improvements. Sequencing bias introduced during pre-analytical and analytical steps requires further improvement especially if NGS is to be used as a method of absolute quantification. Besides improving experimental methods for miRNA profiling, computational prediction tools could facilitate the discovery of miRNA disease biomarkers. In recent years, more databases collecting miRNA-disease association have become available, alongside with a variety of algorithms capable of predicting miRNA biomarkers (reviewed in detail in [34]). The computational prediction tools are typically based on either network algorithms [35] or machine learning [36,37], and particularly the latter approach has shown promise in discovering previously unknown miRNA-disease associations. Combining *in silico* prediction models and accurate experimental miRNA quantification tools, could further enhance future miRNA biomarker discovery.

## Material and methods

### RNA extraction and library preparation

Blood was obtained from healthy volunteers (n = 2) and processed to generate plasma (P) and serum (S) according to standard operating protocols as reported previously [38]. Approvals were obtained from the East London and the City Research Ethics Committee 1 (09/H0703/27). All participants provided written consent. RNA was extracted from 600 µl of plasma or serum using the miRNeasy Serum/Plasma kit (Qiagen) according to the manufacturer’s protocol. Murine brain tissue (B) was obtained from E13.5 mouse embryos (*n *= 2) and RNA was extracted utilising the miRNeasy micro kit (Qiagen). The purified RNA was quantified with Qubit RNA HS assay kit (Thermo Fisher Scientific). The miRXplore Universal Reference (U) containing ~ 950 synthetic miRNAs at equimolar ratio, was utilized as positive control (*n *= 2, technical replicates) (Miltenyi Biotech). All input quantities for small RNA library preparation are indicated in Table 1.

Sequencing libraries were prepared, strictly adhering to the manufacturer’s instructions. Adapter dilution and cycle numbers were set according to the user manual and are listed in Table 1. The size profile of the individual libraries were analyzed utilising D1000 DNA High Sensitivity Screen Tape on a 4200 TapeStation System (both Agilent). Libraries were quantified on a Qubit with the DNA High Sensitivity kit (Life Technologies).

Quantified libraries were mixed at equimolar ratio for each kit and sequenced as follows: Nextflex and NEBNext with a 1 × 51 setup using the NextSeq 500 75-cycle high output sequencing kit and the NextSeq 550 sequencer; SMARTer smRNA-Seq with 1 × 51 setup using the HiSeq rapid SBS kit v4 and HiSeq 2500 sequencer; QIAseq with a 1 × 76 setup utilising MiSeq reagent kit v2 and MiSeq sequencer (all Illumina). Due to differing sequencing requirements (SMARTer smRNA-Seq libraries are not advised to be sequenced on NextSeq, QIAseq require different read length) and incompatible indexes (QIAseq and Nextflex indexes were incompatible) it was not possible to sequence all kits in parallel on the same platform.

### Sequencing data analysis

Raw fastq files have been deposited in the NCBI Sequence Read Archive – accession number SUB3543145. The computational analysis pipeline is outlined in Figure 1(c). In brief, raw reads were quality checked with the FASTQC package (version 0.11.2). Adapters were removed as required for each kit using the cutadapt software (version 1.14) and tolerating 10% error. Reads without adapters or shorter than 17 bases were removed. Of note, the unique molecular indices (UMI) sequences in the QIAseq libraries were trimmed off together with the adapters, so that all kits could be analyzed in parallel utilising the same computational pipeline.

Next, human (*Homo sapiens*, Hg38 assembly) and mouse (*Mus musculus*, mm10 assembly) reference genomes were downloaded from UCSC (http://hgdownload.cse.ucsc.edu/downloads.html) and indexed utilising **bowtie-build** function (version bowtie-1.1.2). The following options were applied for mapping the processed reads from serum, plasma and murine brain to the reference genome: **-v1** (allowing one mismatch) **-m20** (allowing up to 20 multi mapping events) – **best – strata** (only return one alignment with the best alignment score). To map the miRXplore universal reference, no mismatches were allowed (**-v0**) and for convenience, only miRNAs mapping to the human genome were considered. Alignments were stored as sorted BAM files utilising samtools (versions 1.3.1). In case of the universal reference, reads were split in random order into two new fastq files, containing either mapped and unmapped reads using the ‘–all’ and ‘–un’ command. Subsequently, equal numbers of mapped reads were used for downstream analysis.

Sorted alignment files were counted with HTseq (version 0.6.1, intersection-nonempty mode) utilising a customized reference file with the annotations coordinates for ncRNA species of interest for human and mouse respectively. Annotation reference files were generated by extracting the coordinates of human and mouse miRNAs, rRNAs, snRNA, snoRNA, scaRNA, sRNA, miscRNA, vaultRNA, YRNA, Mt- rRNA and Mt- tRNA from the respective annotation file on the Ensembl website (Homo_sapiens.GRCh38.89.chr.gtf.gz and Mus_musculus.GRCm38.89.chr.gtf.gz) using the **grep** command. Next, the genome coordinates for human and mouse tRNAs were extracted from the UCSC table browser (https://genome.ucsc.edu/cgi-bin/hgTables). Human and murine piRNA annotations were obtained from the piRNABank (tp://pirnabank.ibab.ac.in/request.html) [13], and converted to the GRCm38.89 coordinate system using the Ensemble Assembly Converter tool. Finally, all individual non-coding RNA annotation files were combined for each species utilizing the **cat** command. The DESeq2 R package (version 1.16.1) was used to normalize for library size [39]. Of note, reads of related miRNAs which are encoded in distinct genetic loci but correspond to the identical mature miRNA sequence (e.g. miR-486–1 and miR-486–2) were combined for the miRNA signature analysis as their genetic origin typically cannot be inferred during sequencing.

The DREME tool from the MEME suite was used to identify enriched sequence motifs of overrepresented miRNAs in the universal reference [23]. Both the 20 most abundant miRNAs and the subset of these accounting for the top 10% of reads were tested against background control sequences consisting of all mature human miRNA sequences present in the universal reference.

Statistical analysis was carried out with GraphPad Prism. Percentage values were transformed utilising the arcsine function and compared by one-way ANOVA with Bonferroni post hoc test.

### miRNA RT-qPCR

10ng of the miRXplore universal reference was reversed transcribed using the MicroRNA Reverse Transcription Kit (Life Technologies) and miRNA specific hairpin RT primer according to the manufacturer’s instructions (assay IDs: hsa-miR-28-3p (002446), hsa-miR-181a-5p (000480) hsa-miR-199a-5p (000498)). Subsequently, miRNAs were amplified using Small RNA TaqMan assays and TaqMan Universal PCR Master Mix as per manufacturer’s instructions (all Life Technologies). Relative quantities were obtained utilising standard curves.

## References

[1] Bartel DP. MicroRNAs: genomics, biogenesis, mechanism, and function. Cell. 2004;116:281–297. 14744438 10.1016/s0092-8674(04)00045-5

[2] Lawrie CH, Gal S, Dunlop HM, et al Detection of elevated levels of tumour-associated microRNAs in serum of patients with diffuse large B-cell lymphoma. Br J Haematol. 2008;141:672–675. 18318758 10.1111/j.1365-2141.2008.07077.x

[3] Mitchell PS, Parkin RK, Kroh EM, et al Circulating microRNAs as stable blood-based markers for cancer detection. Proc Natl Acad Sci USA. 2008;105:10513–10518. 18663219 10.1073/pnas.0804549105 PMC2492472

[4] Wang J, Chen J, Sen S MicroRNA as biomarkers and diagnostics. J Cell Physiol. 2016;231:25–30. 26031493 10.1002/jcp.25056 PMC8776330

[5] Laterza OF, Lim L, Garrett-Engele PW, et al Plasma MicroRNAs as sensitive and specific biomarkers of tissue injury. Clin Chem. 2009;55:1977–1983. 19745058 10.1373/clinchem.2009.131797

[6] Friedländer MR, Chen W, Adamidi C, et al Discovering microRNAs from deep sequencing data using miRDeep. Nat Biotechnol. 2008;26:407–415. 18392026 10.1038/nbt1394

[7] Neilsen CT, Goodall GJ, Bracken CP IsomiRs–the overlooked repertoire in the dynamic microRNAome. Trends Genet. 2012;28:544–549. 22883467 10.1016/j.tig.2012.07.005

[8] Mestdagh P, Hartmann N, Baeriswyl L, et al Evaluation of quantitative miRNA expression platforms in the microRNA quality control (miRQC) study. Nat Methods. 2014;11:809–815. 24973947 10.1038/nmeth.3014

[9] Git A, Dvinge H, Salmon-Divon M, et al Systematic comparison of microarray profiling, real-time PCR, and next-generation sequencing technologies for measuring differential microRNA expression. RNA. 2010;16:991–1006. 20360395 10.1261/rna.1947110 PMC2856892

[10] Hafner M, Renwick N, Brown M, et al RNA-ligase-dependent biases in miRNA representation in deep-sequenced small RNA cDNA libraries. RNA. 2011;17:1697–1712. 21775473 10.1261/rna.2799511 PMC3162335

[11] Raabe CA, Tang T-H, Brosius J, et al Biases in small RNA deep sequencing data. Nucleic Acids Res. 2014;42:1414–1426. 24198247 10.1093/nar/gkt1021 PMC3919602

[12] Jayaprakash AD, Jabado O, Brown BD, et al Identification and remediation of biases in the activity of RNA ligases in small-RNA deep sequencing. Nucleic Acids Res. 2011;39:e141–e141. 21890899 10.1093/nar/gkr693 PMC3241666

[13] Fuchs RT, Sun Z, Zhuang F, et al Bias in ligation-based small RNA sequencing library construction is determined by adaptor and RNA structure. PLoS One. 2015;10:e0126049. 25942392 10.1371/journal.pone.0126049 PMC4420488

[14] Linsen SEV, de Wit E, Janssens G, et al Limitations and possibilities of small RNA digital gene expression profiling. Nat Methods. 2009;6:474–476. 19564845 10.1038/nmeth0709-474

[15] Baran-Gale J, Kurtz CL, Erdos MR, et al Addressing bias in small RNA library preparation for sequencing: a new protocol recovers MicroRNAs that evade capture by current methods. Front Genet [Internet]. 2015 [cited 2017 11 13];6 Available from: https://www.ncbi.nlm.nih.gov/pmc/articles/PMC4686641/10.3389/fgene.2015.00352 PMC4686641 26734062

[16] Baker M MicroRNA profiling: separating signal from noise. Nat Methods. 2010;7:687–692. 20805796 10.1038/nmeth0910-687

[17] Alon S, Vigneault F, Eminaga S, et al Barcoding bias in high-throughput multiplex sequencing of miRNA. Genome Res. 2011;21:1506–1511. 21750102 10.1101/gr.121715.111 PMC3166835

[18] Bissles S, Wild S, Bosio A Universal reference for microRNA research. Bergisch Gladbach, Germany: Miltenyi Biotec GmbH; 2008.

[19] Bissels U, Wild S, Tomiuk S, et al Absolute quantification of microRNAs by using a universal reference., Absolute quantification of microRNAs by using a universal reference. RNA. 2009;15(15):2375,2375–84. 19861428 10.1261/rna.1754109 PMC2779673

[20] Xie X, Tang F, Yang Z, et al MicroRNA-derived fragment length polymorphism assay. Sci Rep. 2015;5:9356. 25790971 10.1038/srep09356 PMC4366852

[21] Bretschneider M, Busch B, Mueller D, et al Activated mineralocorticoid receptor regulates micro-RNA-29b in vascular smooth muscle cells. FASEB J. 2016;30:1610–1622. 26728178 10.1096/fj.15-271254

[22] Kraus B, Mönk B, Sliva K, et al Expression of human endogenous retrovirus-K coincides with that of micro-RNA-663 and −638 in germ-cell tumor cells. Anticancer Res. 2012;32:4797–4804. 23155245

[23] Bailey TL DREME: motif discovery in transcription factor ChIP-seq data. Bioinformatics. 2011;27:1653–1659. 21543442 10.1093/bioinformatics/btr261 PMC3106199

[24] Freedman JE, Gerstein M, Mick E, et al Diverse human extracellular RNAs are widely detected in human plasma. Nat Commun. 2016;7:11106. 27112789 10.1038/ncomms11106 PMC4853467

[25] Hafner M, Landgraf P, Ludwig J, et al Identification of microRNAs and other small regulatory RNAs using cDNA library sequencing. Methods. 2008;44:3–12. 18158127 10.1016/j.ymeth.2007.09.009 PMC2847350

[26] Tonge DP, Gant TW What is normal? Next generation sequencing-driven analysis of the human circulating miRNAOme. BMC Mol Biol [Internet]. 2016 [cited 2017 12 16];17 Available from: https://www.ncbi.nlm.nih.gov/pmc/articles/PMC4748454/10.1186/s12867-016-0057-9 PMC4748454 26860190

[27] Darnet S, Moreira FC, Hamoy IG, et al High-throughput sequencing of miRNAs reveals a tissue signature in gastric cancer and suggests novel potential biomarkers. Bioinform Biol Insights. 2015;9:1–8. 10.4137/BBI.S23773 PMC4485834 26157332

[28] Wang K, Yuan Y, Cho J-H, et al Comparing the MicroRNA spectrum between serum and plasma. PLoS One. 2012;7:e41561. 22859996 10.1371/journal.pone.0041561 PMC3409228

[29] Leucht C, Stigloher C, Wizenmann A, et al MicroRNA-9 directs late organizer activity of the midbrain-hindbrain boundary. Nat Neurosci. 2008;11:641. 18454145 10.1038/nn.2115

[30] Ling K-H, Brautigan PJ, Hahn CN, et al Deep sequencing analysis of the developing mouse brain reveals a novel microRNA. BMC Genomics. 2011;12:176. 21466694 10.1186/1471-2164-12-176 PMC3088569

[31] Lagos-Quintana M, Rauhut R, Yalcin A, et al Identification of tissue-specific microRNAs from mouse. Curr Biol. 2002;12:735–739. 12007417 10.1016/s0960-9822(02)00809-6

[32] Sorefan K, Pais H, Hall AE, et al Reducing ligation bias of small RNAs in libraries for next generation sequencing. Silence. 2012;3:4. 22647250 10.1186/1758-907X-3-4 PMC3489589

[33] Dhahbi JM, Spindler SR, Atamna H, et al Deep sequencing of serum small RNAs identifies patterns of 5′ tRNA half and YRNA fragment expression associated with breast cancer. Biomark Cancer. 2014;6:37–47. 25520563 10.4137/BIC.S20764 PMC4260766

[34] Chen X, Xie D, Zhao Q, et al MicroRNAs and complex diseases: from experimental results to computational models. Brief Bioinform. 2017. 10.1093/bib/bbx130 29045685

[35] Chen X, Xie D, Wang L, et al BNPMDA: Bipartite Network Projection for MiRNA-Disease Association prediction. Bioinformatics. 2018. 10.1093/bioinformatics/bty333 29701758

[36] Chen X, Huang L, Xie D, et al EGBMMDA: Extreme Gradient Boosting Machine for MiRNA-Disease Association prediction. Cell Death Dis. 2018;9:3. 29305594 10.1038/s41419-017-0003-x PMC5849212

[37] Chen X, Huang L LRSSLMDA: Laplacian Regularized Sparse Subspace Learning for MiRNA-Disease Association prediction. PLoS Comput Biol. 2017;13:e1005912. 29253885 10.1371/journal.pcbi.1005912 PMC5749861

[38] Lu C-H, Macdonald-Wallis C, Gray E, et al Neurofilament light chain. Neurology. 2015;84:2247–2257. 25934855 10.1212/WNL.0000000000001642 PMC4456658

[39] Anders S, Huber W Differential expression analysis for sequence count data. Genome Biol. 2010;11:R106. 20979621 10.1186/gb-2010-11-10-r106 PMC3218662

