# Supplementary material for: Evaluation of methodologies for microRNA biomarker detection by next generation sequencing
Source: RNA Biol. 2018 Sep 18;15(8):1133–45. doi: 10.1080/15476286.2018.1514236 (PMC6161688; doi:10.1080/15476286.2018.1514236)
Supplement: Supplemental Material [file krnb-15-08-1514236-s001.docx]

**Supplementary Figures**


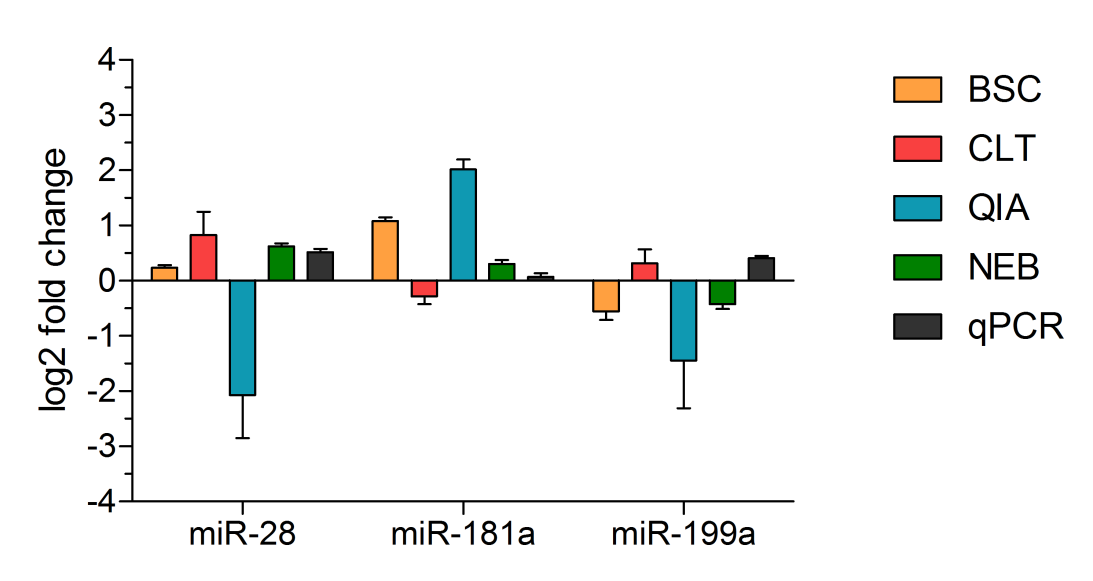


**Figure S1**

Comparison of miXplore sequencing results and RT-qPCR validation for three selected miRNAs. The graph shows the log2 fold change of detected miRNA expression compared to the mean expression. Values are mean +SD.


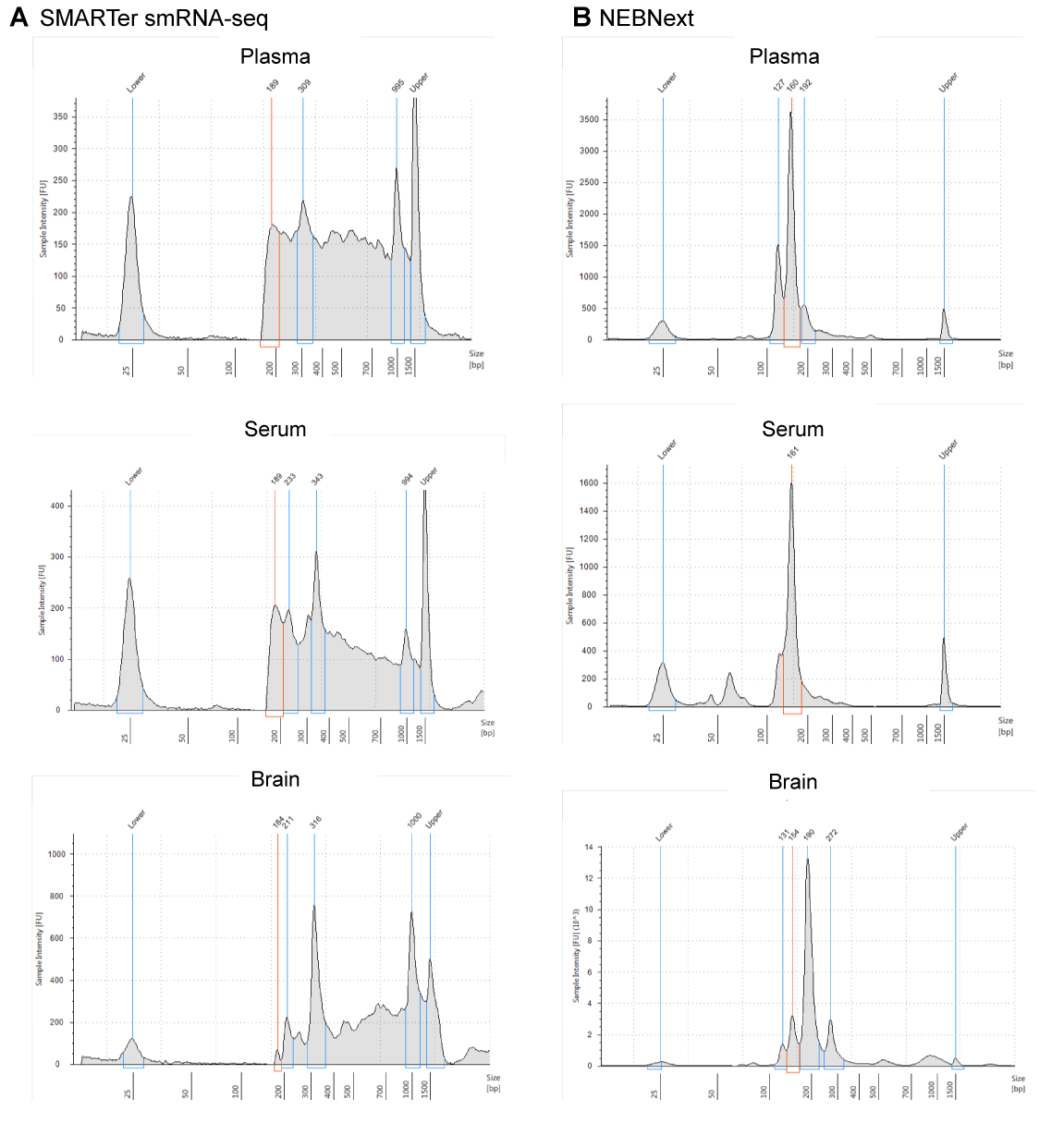


**Figure S2 TapeStation profile of SMARTer smRNA-Seq and NEBNext libraries prior to gel size selection**

Small RNA libraries were prepared according to the manufacturer’s instructions and 1 µl pf each sample was analysed with a High Sensitivity D1000 ScreenTape on a 4200 TapeStation system (both Agilent) prior to size selection on the gel. An example for plasma, serum and brain is indicated for the two kits without bead based purification. The peak predicted to correspond to miRNAs plus adapters is highlighted in orange. Libraries prepared with 3’ tailing methodology (SMARTer smRNA-Seq) result in the presence of a large variety of RNA species with variable sizes. In contrast, more defined small RNA peaks are visible in the libraries prepared with NEBNext. Peaks ~20 nt shorter than the predicted miRNA peak are likely to correspond to adapter dimers.


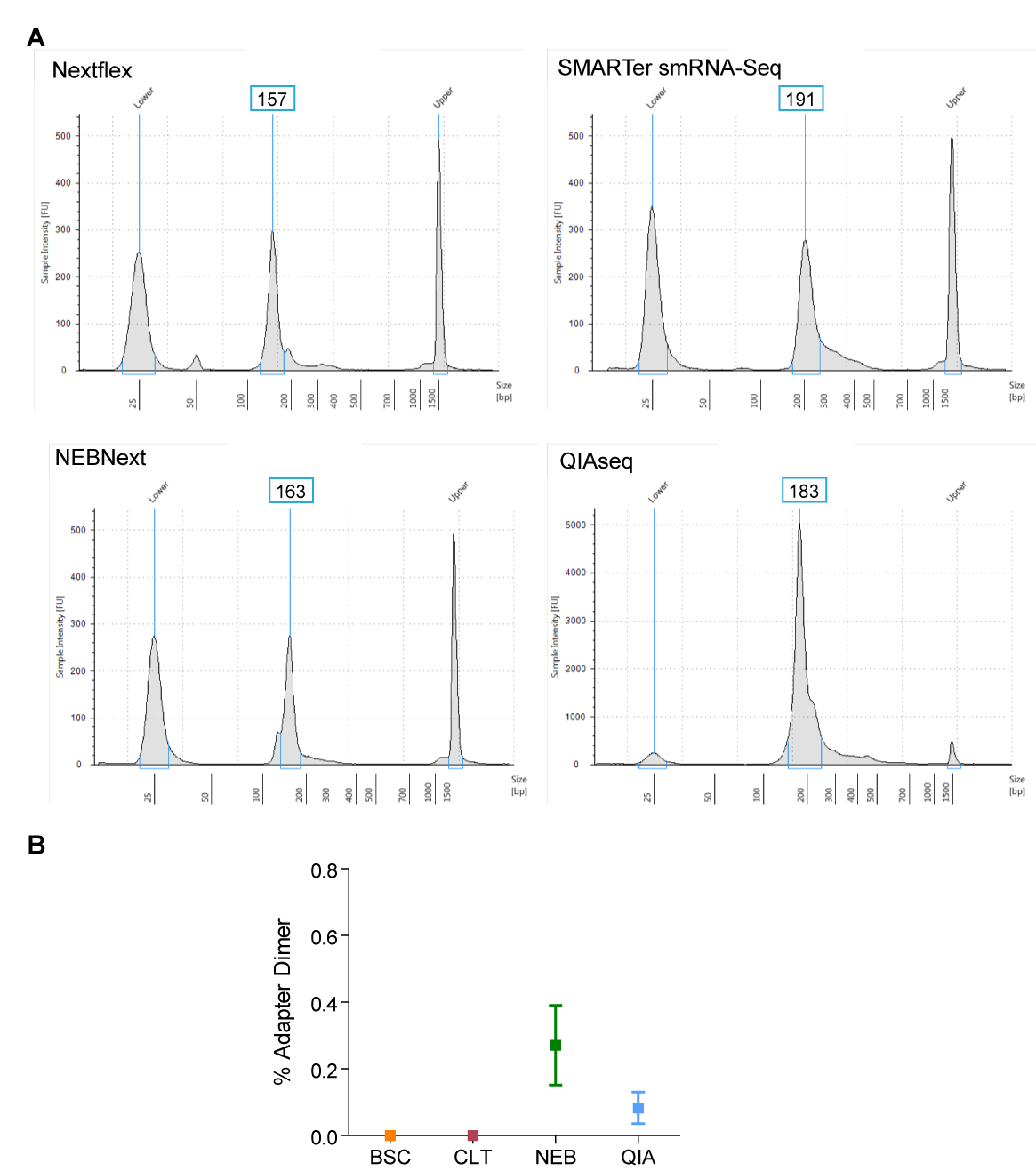


**Figure S3 TapeStation profile of size-selected pooled libraries for each kit.**

Small RNA libraries were prepared from the miRXplore universal reference, plasma, serum brain tissue according to the manufacturers’ instructions. Following size selections libraries were pooled in an equimolar ratio for each kit and 2 µl where analysed on a D1000 High Sensitivity ScreenTape. Expected sizes for miRNA and adapters were as follows: ~150 bp for Nextflex, ~176 for SMARTer smRNA-Seq, ~147 for NEBNext and ~173 for QIAseq. Due to salt levels in library solvents the observed sizes can be higher than expected. All library preparation protocols resulted in the presence of RNA species larger than miRNA (high molecular weight ‘contamination’ in the TapeStation profile). Peaks ~20 nt shorter than the predicted miRNA peak are likely to correspond to adapter dimers. (B) Percentage of reads mapping to adapters compared to total reads. Values +/- SEM.


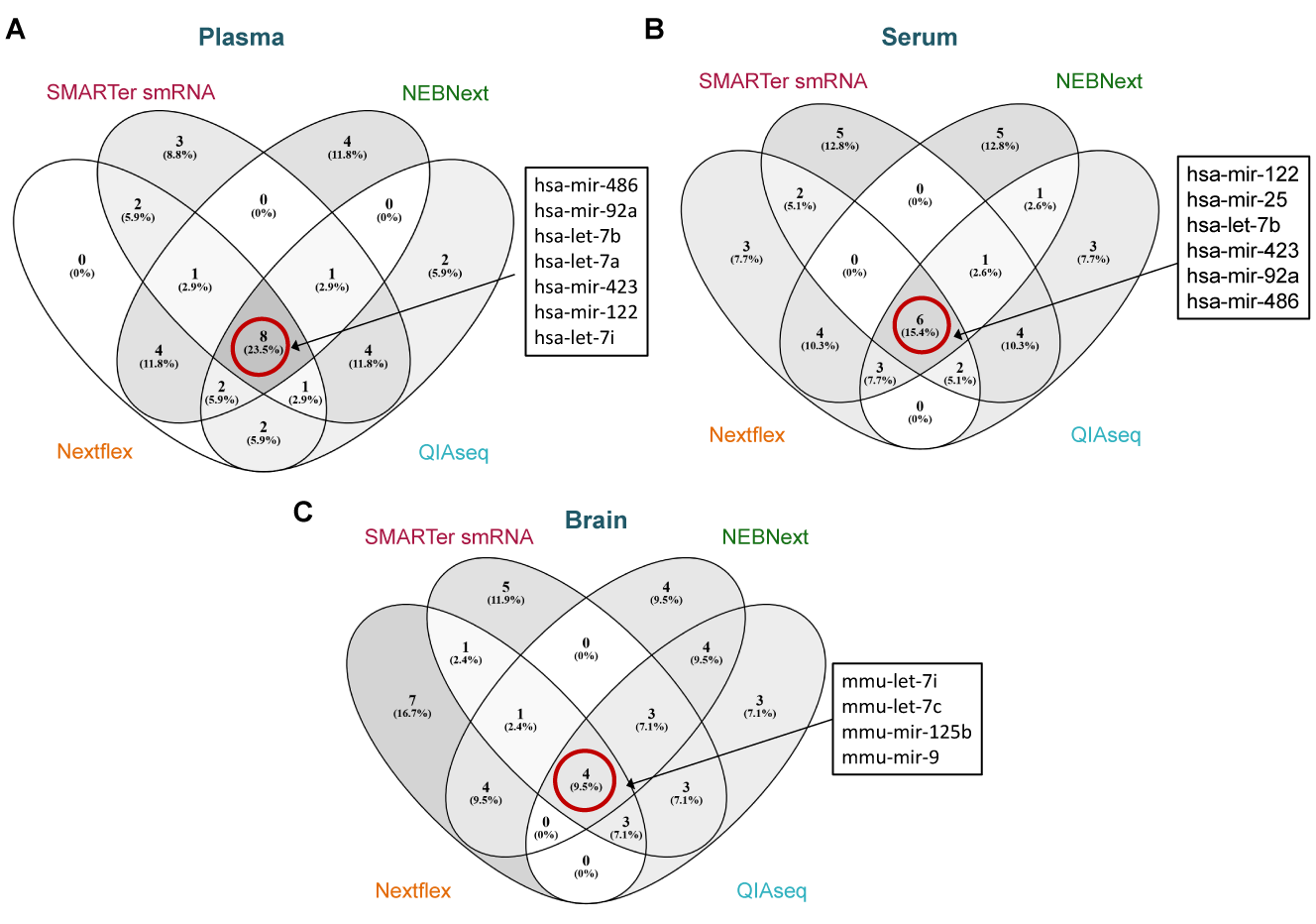
**Figure S4 Overlap of the miRNA signatures in human plasma, human serum and murine brain.**

The overlap of the 20 most abundant miRNAs (utilising the mean percentage frequency) between each methodology are indicated as Venn diagrams for (A) human plasma, (B) human serum and (C) murine brain. The list indicates miRNAs that ranked among the 20 most abundant miRNAs for all kits.

**Supplementary Tables**

**Table S1** **Summary of HTSeq counting statistics and correlation analysis for the miRXplore universal reference.**

Counting statistics of the miRXplore universal reference. BSC and NEB were sequenced utilising NextSeq sequencer, CLT with a HiSeq 2500 sequencer and QIA on MiSeq (all Illumina). No mismatch tolerated during bowtie mapping (-v0 option) and reads mapping more than 20 times to the genome were discarded (-m20 option).

**Table S2 Summary of library size, bowtie mapping and HTseq counting statistics for human plasma samples.**

BSC and NEB were sequenced utilising NextSeq sequencer, CLT with a HiSeq 2500 sequencer and QIA on MiSeq (all Illumina). One mismatch tolerated during bowtie mapping (-v1 option). Reads mapping more than 20 times to the genome were discarded (-m20 option). The -m20 option filtered out a considerable amount of reads in samples prepared with the SMARTer smRNA (CLT) kit. Sample biotype composition was calculated in percentage compared to total mapped reads. Libraries prepared with Nextflex (BSC) or QIAseq (QIA) were most enriched in miRNA reads. BSC: Bioo Scientific; Clontech: CLT; NEB: New England Biolabs; QIA: Qiagen

**Table S3 Summary of library size, bowtie mapping and HTseq counting statistics for human serum samples.**

BSC and NEB were sequenced utilising NextSeq sequencer, CLT with a HiSeq 2500 sequencer and QIA on MiSeq (all Illumina). The bowtie v1 option refers to one tolerated mismatch during mapping. -m option was set to 20, so reads that mapped more than 20 times to the genome were discarded. The -m20 option filtered out a considerable amount of reads in samples prepared with the SMARTer smRNA-Seq (CLT) kit. Sample biotype composition was calculated in percentage compared to total mapped reads. Serum libraries prepared with QIAseq kit (QIA) contained the highest percentage of reads mapping to miRNAs. BSC: Bioo Scientific; Clontech: CLT; NEB: New England Biolabs; QIA: Qiagen

**Table S4 Summary of library size, bowtie mapping and HTseq counting statistics for murine brain samples.**

BSC and NEB were sequenced utilising NextSeq sequencer, CLT with a HiSeq 2500 sequencer and QIA on MiSeq (all Illumina). One mismatch was tolerated during bowtie mapping (-v1 option). Reads mapping more than 20 times to the genome were discarded (-m20 option). Sample biotype composition was calculated in percentage compared to total mapped reads. Libraries prepared with the NEBNext protocol were most enriched in miRNAs. For the BSC kit, more than 99% of the reads in the ‘other sRNA category’ were snoRNA mapping reads (data not shown). BSC: Bioo Scientific; Clontech: CLT; NEB: New England Biolabs; QIA: Qiagen
